# A new ant species of the genus *Tapinoma* (Hymenoptera, Formicidae) from Saudi Arabia with a key to the Arabian species

**DOI:** 10.3897/zookeys.212.3325

**Published:** 2012-07-30

**Authors:** Mostafa R. Sharaf, Abdulrahman S. Aldawood, Magdi S. ElHawagry

**Affiliations:** 1 Plant Protection Department, College of Food and Agriculture Sciences, King Saud University, Riyadh 11451, PO Box 2460, Kingdom of Saudi Arabia; 2 Basic Sciences Department, Community College, Al-Baha University, Al-Baha, P. O. Box 1598, Kingdom of Saudi Arabia, Project: Survey and Classification of Agricultural and Medical Insects in Al-Baha Province

**Keywords:** Dolichoderinae, Kingdom of Saudi Arabia, Middle East, new species, Palaearctic, Al Sarawat Mountains

## Abstract

*Tapinoma wilsoni* **sp. n.** is described and illustrated from Saudi Arabia based on the worker caste collected from Al Baha, Saudi Arabia. It closely resembles *Tapinoma lugubre* Santschi 1917, from Zimbabwe in body measurements but can be distinguished from the latter species by the yellowish brown color; the yellowish pubescence, the two pairs of hairs present on the anterior part of the head; and the distinctly concave anterior clypeal margin. Biological and ecological notes of the new species are presented. An identification key to the workers of the Arabian species of the genus *Tapinoma* is given.

## Introduction

The genus *Tapinoma* was established by Foerster (1850), with the type-species *Tapinoma collina* Foerster. The genus currently comprises 126 described species and subspecies (Bolton et al. 2007) distributed worldwide in tropical and temperate regions (Brown 2000). Members of this genus are generalized foragers (Brown 2000), nesting in a wide variety of habitats, ranging from grasslands, open fields, woodlands, to inside buildings. The majority of species nest in the ground under objects such as stones or tree logs, other species build nests under bark of logs and stumps, in plant cavities, insect galls or refuse piles (Smith 1965). *Tapinoma simrothi* Krausse in Saudi Arabia was observed nesting in a decaying carpet, among roots of graminae plants and attending unidentified mealybugs, and also coexisting with *Solenopsis saudiensis* Sharaf & Aldawood (Sharaf and Aldawood 2011).

Terminology used to characterize *Tapinoma* follows Bolton (1973, 1994): Mandibles with two or three large apical teeth, followed by a row of denticles; palp formula 6,4; clypeus with or without median anterior border emarginated; antennae 12-segmented; propodeum unarmed or rarely with a pair of blunt tubercles; petiole reduced or vestigial, overhung by the first gastral segment and not visible in dorsal view; only four gastral segments visible in dorsal view; fifth tergite reflexed below the fourth, visible in ventral view where it forms a transverse plate abutting the fifth sternite; the anal and associated orifices are thus situated ventrally.

Only two named species of *Tapinoma* have been recorded from countries occupying the Arabian Peninsula, *Tapinoma melanocephalum* (Fabricius) from Saudi Arabia and Oman (Collingwood 1985), UAE (Collingwood et al. 1997, Collingwood et al. 2011),Yemen (Collingwood and Agosti 1996, Collingwood and Van Harten 2001) and Socotra Archipelago (Collingwood et al. 2004); *Tapinoma simrothi* from Saudi Arabia (Collingwood 1985), Kuwait (Collingwood 1985, Collingwood and Agosti 1996), UAE (Collingwood et al. 1997, Collingwood et al. 2011), Qatar (Sharaf and Abdeldayem, in preparation), Oman (Collingwood 1988) and Yemen (Collingwood and Agosti 1996).

In the present study, a new species of the genus *Tapinoma* is described from Saudi Arabia and a key to the known Arabian species is given.

## Materials and methods

Measurements and indices follow Bolton (2007).

### Measurements

**Total Length (TL):** The total outstretched length of the ant from the mandibular apex to the gastral apex.

**Head Length (HL):** The length of the head capsule, excluding the mandibles; measured in full-face view in a straight line from the mid-point of the anterior clypeal margin to the mid-point of the posterior margin. In species where one or both of these margins are concave, the measurement is taken from the mid-point of a transverse line that spans the apices of the projecting portions.

**Head Width (HW):** The maximum width of the head behind the eyes, measured in full-face view.

**Scape Length (SL):** The maximum straight-line length of the scape, excluding the basal constriction or neck that occurs just distal of the condylar bulb.

**Pronotal Width (PW):** The maximum width of the pronotum in dorsal view.

**Weber’s length of Mesosoma (WL):** The diagonal length of the mesosoma in profile, from the most anterior point of the pronotum to the posterior basal angle of the metapleuron.

All measurements are expressed in millimeters.

### Indices

**Cephalic Index (CI):** HW divided by HL, × 100.

**Dorsal Thoracic Index (DTI):** In dorsal view, the length from the mid-point of the anterior pronotal margin to the midpoint of the metanotal groove, divided by PW, × 100.

**Eye Position Index (EPI):** In full-face view the straight-line length (parallel to the long axis of the head) from the most anterior point of the eye to the anterior clypeal margin, divided by the straight-line length from the most posterior point of the eye to the posterior margin, × 100.

**Ocular Index (OI):** Maximum diameter of eye divided by HW, × 100.

**Scape Index (SI):** SL divided by HW, × 100.

### Illustrations

Specimens were photographed by using Digital color images that were created using a Leica DFC 425 camera in combination with the Leica Application Suite software (version 3.8). All images presented are available online at AntWeb (http://www.antweb.org).

### Depositories of type specimens

BMNH Natural History Museum, London, United Kingdom.


CASC California Academy of Sciences Collection, San Francisco, California, USA.


KSMA King Saud Museum of Arthropods, King Saud University, Riyadh, Kingdom of Saudi Arabia.


MCZC Museum of Comparative Zoology, Cambridge, MA, USA.


MHNG Muséum ďHistoire Naturelle, Geneva, Switzerland.


NHMB Naturhistorisches Museum, Basel, Switzerland.


WMLC World Museum Liverpool, Liverpool, United Kingdom.


SEMC Division of Entomology (Snow Entomological Collections), University of Kansas Natural History Museum, Lawrence, Kansas, USA.


## Results

### Tapinoma wilsoni

Sharaf & Aldawood sp. n.

urn:lsid:zoobank.org:act:2680E437-3109-4F74-B21D-5A99A8ABB8C0

http://species-id.net/wiki/Tapinoma_wilsoni

Figs 1 [Fig F2] 3

#### Holotype worker.

Saudi Arabia, Al Baha, Al Sarawat Mountains, Dhi Ayn Archaeological Village, 19.92972^o^N, 41.44278^o^E, 741 m, 15.v.2011 (*M. R. Sharaf Leg*.); deposited in the King Saud Museum of Arthropods, College of Food and Agriculture Sciences, King Saud University, Riyadh, Kingdom of Saudi Arabia.

#### Paratype workers.

29 workers, same locality as holotype, deposited as follows: 1 in **MHNG** (Dr Bernhard Merz); 1 in **NHMB** (Mrs. Isabelle Zürcher-Pfander); 1 in **CASC** (Dr Brian Fisher); 1 in **MCZC** (Dr Stefan Cover); 2 in **WMLC** (Tony Hunter), 1 in **BMNH** (Mr. Barry Bolton); 1 in SEMC (Prof. Michael S. Engel) the remaining specimens in **KSMA** (M. R. Sharaf).

#### Additional paratype workers.

Saudi Arabia, Al Baha, Dhi Ayn Archaeological Village, 19.92976°N, 41.44187°E ± 50 m, 23.ix.2011 *(B.L. Fisher Leg.)* 4 deposited in **CASC.**

#### Measurements.

**Holotype**: TL: 1.84, HL: 0.51, HW: 0.41, SL: 0.46, PW: 0.29, WL: 0.56, EL: 0.11. Indices: CI: 80, SI: 112, OI: 27, EPI: 71, DTI: 134.

**Paratypes:** TL: 1.56-1.84, HL: 0.49-0.53, HW: 0.36-0.42, SL: 0.35-0.51, PW: 0.25-0.31, WL: 0.49-0.63, EL: 0.09-0.14. Indices: CI: 73-84, SI: 105-133, OI: 22-33, EPI: 67-82, DTI: 114-145 (11 measured).

#### Description of worker.

Head distinctly longer than broad with feebly convex posterior margin and sides; anterior clypeal margin broadly and distinctly concave; scapes, in full-face view, surpassing posterior margin of head by about 1/6 of its length; all funicular segments clearly longer than broad; eyes relatively large (OI 22-33) with 8 ommatidia in the longest row; mandibles large, armed with two distinct apical teeth followed by two smaller teeth, the third tooth being smaller than the fourth; the remaining masticatory margin equipped with several indistinct and tiny denticles; mandibles with several long yellow hairs; head pilosity a fine, whitish, appressed pubescence; promesonotum in profile straight or feebly convex; metanotal groove indistinct; propodeum in profile with the transition from dorsum to declivity sharply defined, the declivity concave and the angle with a raised apex; body pilosity restricted to two pairs of setae on dorsum of head, located close to antennal insertions and at the level of the anterior eye margin, none on mesosoma, one pair on second and third gastral tergites (absent in some individuals), apex of gaster with several pairs of long hairs; body very finely and densely shagreenate; mesosoma dull, head and gaster more or less shining. Color brownish yellow or yellowish with very faint brownish tint on dorsum of head, appendages clear yellow.

#### Etymology.

A patronymic name honors Prof. Edward O. Wilson in recognition of his valuable contribution to the science of myrmecology over several decades.

The following key separates the members of the genus *Tapinoma* hitherto known from the Arabian Peninsula, including *Tapinoma wilsoni*.

**Figure 1.**
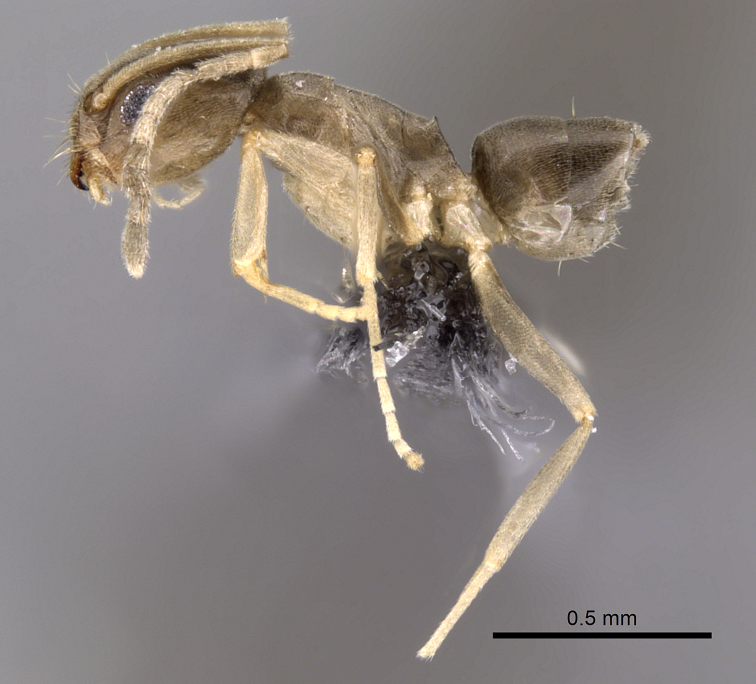
Lateral view of paratype worker of *Tapinoma wilsoni* sp. n. (CASENT0263919)

**Figure 2. F2:**
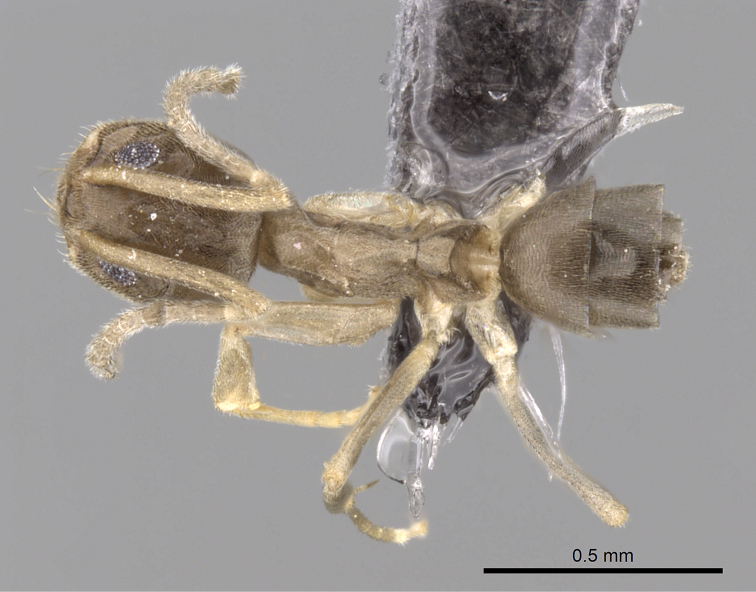
Dorsal view of paratype worker of *Tapinoma wilsoni* sp. n. (CASENT0263919)

**Figure 3.**
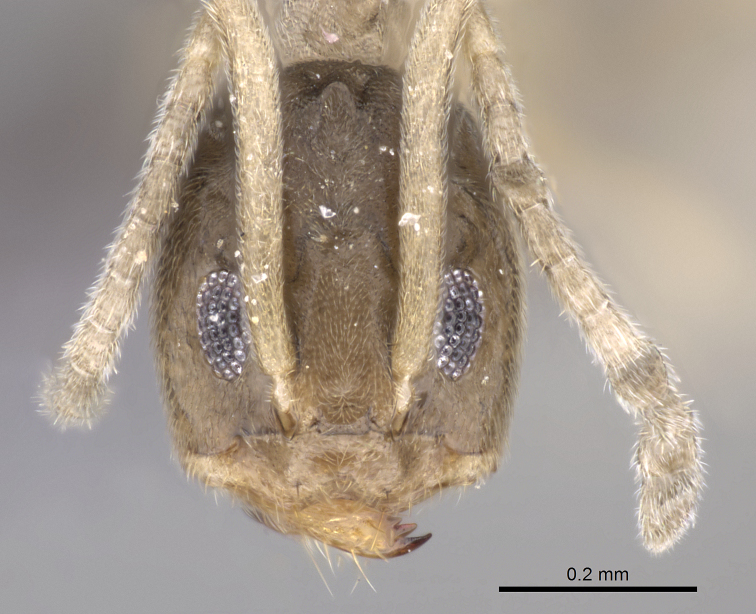
Frontal view of paratype worker of *Tapinoma wilsoni* sp. n. (CASENT0263919)

##### Key to the Arabian species of the genus *Tapinoma*

**Table:** 

1	Larger species, TL more than 2.0 mm; color darker, uniformly blackish brown or black	*Tapinoma simrothi* Krausse
–	Smaller species, TL less than 2.0 mm; uniformly pale yellow or brownish yellow, or at least with gaster yellow	2
2	Propodeum in profile with the transition from dorsum to declivity sharply defined, the declivity concave and the angle with a raised apex; brownish yellow or yellowish with very faint brownish tint on head dorsum, appendages clear yellow	*Tapinoma wilsoni* sp. n.
–	Propodeum in profile with the transition from dorsum to declivity no more than a rounded angle; head and mesosoma dark yellowish-brown, gaster yellow	*Tapinoma melanocephalum* (Fabricius)

## Discussion

*Tapinoma wilsoni* appears most similar to *Tapinoma lugubre* Santschi, 1917, which was described from Zimbabwe. The two species are similar in size (TL 1.50–1.80) and both have the propodeum in profile with the transition from dorsum to declivity sharply defined, the declivity concave, and the angle with a raised apex. *Tapinoma wilsoni* can be easily separated from *Tapinoma lugubre* by the following characters: color brownish yellow or yellowish with fine brownish tint on head dorsum, appendages clear yellow, while *Tapinoma lugubre* is much darker brown or dull yellowish black, with occiput and gaster blackish. In *Tapinoma wilsoni*, the scape surpasses the posterior margin of head by about 1/6 of its length, whereas in *Tapinoma lugubre*, the scape surpasses the posterior margin of head by about 1/4 not similar in formatting to 1/6 in the above line!. In addition, *Tapinoma wilsoni* has theanterior clypeal margin distinctly concave, while in *Tapinoma lugubre* the middle of the anterior clypeal margin is shallowly concave. Moreover, *Tapinoma wilsoni* has two pairs of setae on the dorsum of the head, one close to antennal insertions and the other at the level of the anterior margin of eyes and several pairs on clypeus; whereas *Tapinoma lugubre* lacks hairs on the dorsum of the head. *Tapinoma wilsoni* can be easily separated from other Arabian species by the concave propodeal declivity and the well-defined angle between dorsum of propodeum and declivity.

**Figure 4.**
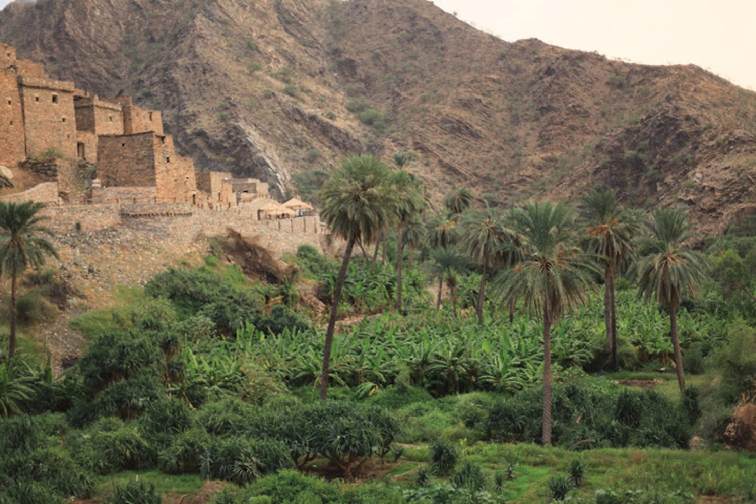
Type Locality Dhi Ayn Archaeological Village, Al-Baha Province, Saudi Arabia. (Brian Fisher photo)

**Habitat and biology.** The specimens of *Tapinoma wilsoni* were found foraging on the ground, and coexisting with the ant species *Carebara abuhurayri* Sharaf & Aldawood, *Tetramorium sericeiventre* Emery, *Pheidole minuscule* Bernard, *Pheidole* sp., *Monomorium destructor* (Jerdon), *Monomorium exiguum* (Forel), *Monomorium* sp., and *Crematogaster* sp. The type locality (Fig. 4) of this new species is a semi isolated area which is completely surrounded by high mountains and largely under banana cultivation. The new species was collected at the base of banana trees. Due to continuous irrigation of the banana plantations, the soil is moist throughout the year. The type locality still has a diversity of native plants as well as many other cultivated species, especially date palm, *Ficus* trees, alfalfa and some lemon trees. Numerous small streams drain this area.

*Tapinoma wilsoni* is the first new species to be described in the genus since Collingwood’s (1985) review of the Arabian ant fauna. We believe that the Mountains of Al Sarwat and Asir (southwestern region of the Arabian Peninsula) may yield a wealth of undescribed ant species.

## Supplementary Material

XML Treatment for Tapinoma wilsoni
